# Resolutions on the Death of Dr. Knight, Dr. Charles Hooker, and Dr. Benjamin Silliman

**Published:** 1865-07

**Authors:** 


Dr. Hooker, of Connecticut, from a special committee, reported the following resolutions on the death of Dr. Knight, Dr. Charles Hooker, and Dr. Benjamin Silliman, all of Connecticut. The report was accepted, and the resolutions adopted:

*Resolved*, That in the death of Dr. Jonathan Knight, twice a President of this Association, the duties of which office he discharged with extraordinary ability, the Medical Profession has lost one of its brightest ornaments; an eminent surgeon, who was conservative in the true sense of that term; a teacher, who by his transparent clearness and terse eloquence, unsurpassed among medical men in this country, exerted through fifty years a wide influence upon the character of the American medical mind; a Christian gentleman, whose genial qualities won for him, to an extent seldom witnessed, the affections not only of intimate friends, but of all those who knew him.

*Resolved*, That in lamenting the sudden departure from this life of Dr. Chas. Hooker, at the height of his usefulness, we cherish the memory of one whose independent mind and restless activity of Christian faithfulness obtained for him great eminence, both as a physician and teacher of medicine; and we call to mind with special pleasure, his earnest devotion from the outset to the interests of this Association.

*Resolved*, That we revere the memory of Professor Benjamin Silliman, who as a leader in the diffusion of science in this country, and in other countries also, was one of the great benefactors of the race, and who by his urbanity, kindness of heart, and cheerful piety, enhanced to an uncommon degree his influence upon the community as a man of science.

Dr. Furman, of New York, presented resolutions adopted by the State Medical Society of New York, and referred to the National Medical Society, against newspaper advertising, except merely the card of the physician.

Dr. Julius Homberger, of New York, Chairman of the Committee on Specialties and Specialists, presented an elaborate individual report, favoring advertising by specialists. The report was laid upon the table for the present, and a second report was submitted by Dr. H. R. Storer, of Boston, also a member of the committee, defending specialists.

Dr. D. Humphreys Storer spoke concerning a passage in the report relating to himself, denying that he was ever a specialist. Dr. H. R. Storer, in reply, defended his own ground. This report was also laid upon the table, temporarily.

Dr. W. Hooker, of Connecticut, another member of the committee, reported that he had not had sufficient time to investigate the report of Dr. Homberger, and therefore could not give an opinion concerning it. With the report of Dr. H. R. Storer he could not entirely agree, especially in that part of the report which referred to specialists as the leaders of the profession.

The reports were then taken from the table for consideration.

Dr. Martin made a motion that the reports be referred back to the committee for consideration another year, and supported his motion at some length.

Dr. Bissell, of New York, moved to amend the previous motion, and moved that the reports be referred to the Committee on Medical Ethics.

Dr. Toner, of Washington, criticised the reports in a severe manner.

Dr. Homberger, of New York, defended his position, and stated as the reason why he did not send his report to the other members of the committee at an earlier date, that he knew they would not agree with him and would not sign it. His remarks were not received very favorably by the members.

Dr. Mayburry, of Pennsylvania, favored strongly the referring of the reports to the Committee on Medical Ethics.

The question was then taken on referring the reports to the Committee on Ethics, and decided in the affirmative. On motion of Dr. Twitchell, of New Hampshire, the Committee on Medical Ethics was instructed to report some definite action on this subject.

The convention adjourned at half-past 12 o'clock.

*The Excursion down the Harbor.*—The gentlemen of the Association, immediately after the adjournment of the business meeting at the State House, proceeded in a body to Commercial Wharf, where they embarked in the steamers Rose Standish and Russia, for the purpose of taking an excursion down the harbor, by invitation of the City Council. On board of the Rose Standish, besides the members of the Medical Society, were His Honor the Mayor, and several members of the City Council, part of the Committee of Arrangements, Chief of

Police Kurtz, a number of invited guests, and Gilmore's full military band in their new uniform. On the Russia, the Brigade Band was stationed. The party on both steamers numbered nearly, if not quite, 1000 people.

The two steamers proceeded down the channel side-by-side. As the party passed the revenue cutter Pawtuxet, they were received with a salute, and the hoisting of the flag. The steamers acknowledged the compliment by dipping their colors and sounding their whistles. The boys on the Massachusetts, school ship, filling the deck and rigging, cheered the excursion lustily and run up the colors. This compliment was also acknowledged by the Rose Standish and the Russia, and the cheers returned by the passengers.

The first landing of the party was at Deer Island, where they were received by Hon. Moses Kimball, Chairman of the Committee on Public Institutions, and were invited by that gentleman to walk through the House of Industry, and examine it as much as they desired. After about a half-hour's time had been consumed in visiting the island, the party reëmbarked, and proceeded further down the harbor.

By the kind permission of Major H. A. Allen, of the Second United States Artillery, commanding the post at Fort Warren, the excursionists were allowed to land, but were requested by the Major to have no conversation with the prisoners. The United States officers at Fort Warren were very attentive to the guests, and extended to them, during their short stay, every attention that was in their power. Major Joel Severns, of the U.S. Volunteers, is post-surgeon.

About four o'clock, the party reached Long Island, and disembarked. In the "Cottage," and in the tents on either side, tables were spread with all the delicacies of the season, and to this attractive feature the attention of the gentlemen was directed immediately upon landing.

After the very pleasant duty of relieving the tables of their burden had been performed, His Honor the Mayor mounted a chair, and calling the gentlemen to order, addressed the company in a speech full of the most cordial hospitality and generous appreciation of the standing and value of the medical profession. We regret that want of space prevents our publishing his remarks in full.

